# Extraordinary terahertz absorption bands observed in micro/nanostructured Au/polystyrene sphere arrays

**DOI:** 10.1186/1556-276X-7-657

**Published:** 2012-11-28

**Authors:** Guotao Duan, Fuhai Su, Wen Xu, Cunlin Zhang, Weiping Cai

**Affiliations:** 1 Key Laboratory of Materials Physics, Institute of Solid State Physics, Chinese Academy of Sciences, Hefei, 230031, China; 2 Department of Physics, Yunnan University, Kunming, 650091, China; 3 Beijing Key Laboratory for Terahertz Spectroscopy and Imaging, Key Laboratory of Terahertz Optoelectronics, Department of Physics, Capital Normal University, Beijing, 100048, China

**Keywords:** Terahertz, Micro/nanostructure, Absorption bands, Quantum confinement

## Abstract

Terahertz (THz) time-domain spectroscopy is carried out for micro/nanostructured periodic Au/dielectric sphere arrays on Si substrate. We find that the metal-insulator transition can be achieved in THz bandwidth via varying sample parameters such as the thickness of the Au shell and the diameter of the Au/dielectric sphere. The Au/polystyrene sphere arrays do not show metallic THz response when the Au shell thickness is larger than 10 nm and the sphere diameter is smaller than 500 nm. This effect is in sharp contrast to the observations in flat Au films on Si substrate. Interestingly, the Au/polystyrene sphere arrays with a 5-nm-thick Au shell show extraordinary THz absorption bands or metallic optical conductance when the diameter of the sphere is larger than 200 nm. This effect is related to the quantum confinement effect in which the electrons in the structure are trapped in the sphere potential well of the gold shell.

## Background

In recent years, artificially structured metal-dielectric systems have attracted extensive attentions due to potential applications in electronic and optoelectronic devices. These advanced material systems have been proposed as a new generation of optic and optoelectronic materials and devices working in terahertz (THz) bandwidth, motivated by realizing and developing new devices that can fill the THz gap [1,2]. For instance, metamaterials composed of subwavelength split-ring resonate arrays allow both electric- and magnetic-dipole interactions, which is promising in the development of novel THz devices such as perfect lens, cloak, etc. [3,4]. On the other hand, metal-dielectric interfaces with the structure of a periodic metallic pattern can give rise to the formation of THz photonic bands via strong coupling between electromagnetic waves and surface plasmon modes [5,6]. An extraordinary light transmission due to surface plasmon polariton (SPP) excitation in THz-frequency domain has been observed in periodic metallic hole arrays [7-10]. Generally, both metamaterials and SPP devices are able to realize the resonant enhancement of the electromagnetic interactions at selective frequency. In traditional two-dimensional planar metallic structures on dielectric substrates, the unit size is normally on subwavelength distance scale and the thickness of the metal film is of the order of about 100 nm, i.e., the size of the metallic unit or lattice is comparable to the coherent length of corresponding photons but far beyond that of conducting electrons. In principle, when the thickness of a metallic film approaches the nanometer distance scale or smaller than the mean free path of electron motion, the electronic conduction can be reduced pronouncedly and, thus, the metal-insulator transition can be observed. Hence, the nanometer-sized metallic structures are not appropriate for electromagnetic devices which can be functional in the manner of metamaterials or SPP devices. This is due mainly to the poor electronic conduction tending to decrease the electric and magnetic interactions. With the rapid development of nanotechnology, now it has become possible to fabricate different nanosized array structures with different materials. This can provide us with new material and device systems to examine light-induced metal-insulator transition and with more freedom to modulate the light response of the material and device systems through varying the array structure artificially. In this paper, we present a systematic study on how metal nanosphere arrays can respond to THz light fields. We would like to demonstrate that THz anomalous absorption bands can be observed in Au/dielectric sphere array structures with a micrometer sphere diameter and a nanometer shell thickness. We intend to find out how the THz response of the sphere arrays differs from those observed in the metamaterials and SPP devices.

## Methods

Polystyrene (PS) spheres with diameters of 200, 500, and 1,000 nm were, respectively, coated on high-resistance Si wafers and self-organized into an orderly colloidal PS monolayer with a structure of hexagonal closest packing [11]. The PS sphere arrays with Au shells were processed by depositing gold onto the PS film by means of ion-beam sputtering [11]. The Au/PS spheres with gold shell thicknesses of 5, 10, and 15 nm were fabricated in the present study. The details of the sample fabrication were documented in [11].

**Figure 1:**
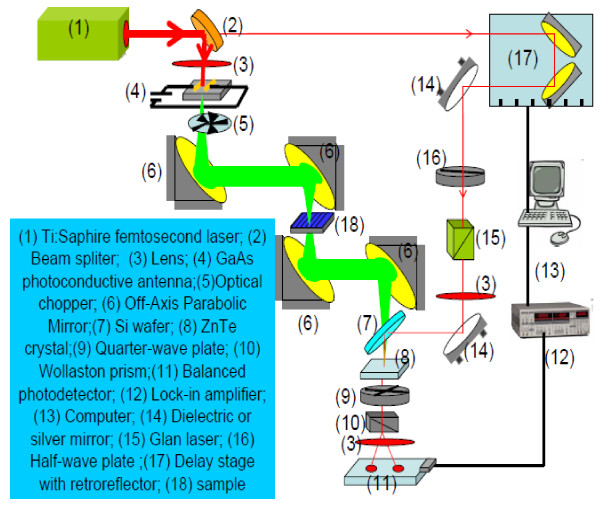
The experimental setup for the THz time-domain spectroscopy measurement.

THz time-domain spectroscopy (TDS) was performed using the standard configuration for the measurement of the THz transmission spectrum (see Figure 1). Here, (1) femtosecond laser pulses are employed as pumping light source, (2) pumping laser pulses are focused on GaAs photoconductive antenna which provides the broadband and pulsed THz radiation source, (3) the free-space electro-optic sampling via ZnTe crystal is applied for detection of light transmission in time domain, and (4) Fourier transformation of the measured data is carried out to obtain the THz transmission spectrum. In the present study, the THz TDS measurements were undertaken under a dry nitrogen purge at room temperature.

## Results and discussion

**Figure 2:**
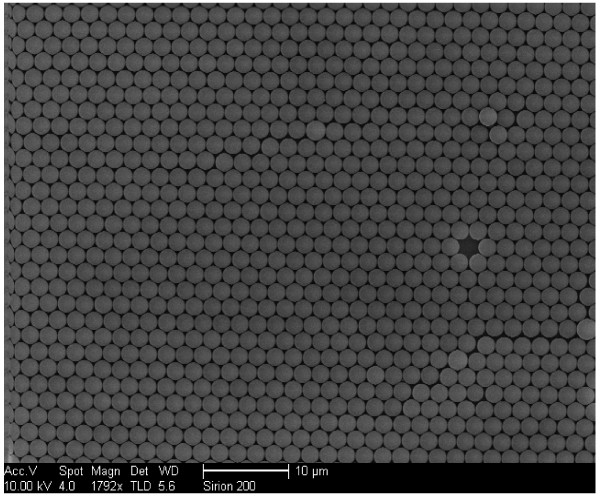
The typical SEM image of micro/nanostructured periodic Au/polystyrene sphere array structure.

**Figure 3:**
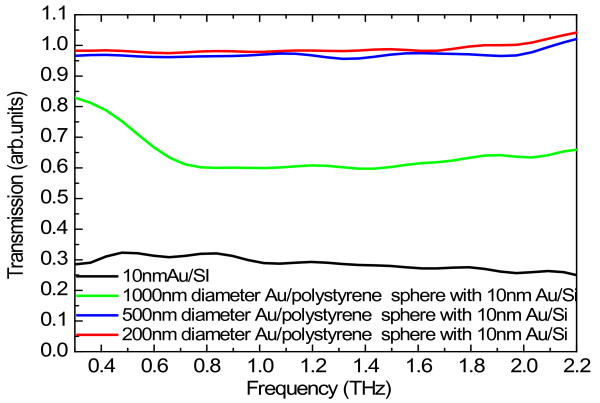
Normalized transmission spectra for different-sized Au/PS sphere arrays with 10-nm-thick Au shell.

The scanning electron microscopy (SEM) image in Figure 2 shows the morphology of the sample used in the investigation. It can be seen that the Au/PS spheres are packed closely to each other with a hexagonal structure and formed nicely an array structure. Further SEM study indicates that the Au nanoclusters formed on the PS spheres are quite uniform as shown in Figure 2. One of the advantages of using Au/PS sphere arrays as an optoelectronic device is that the optoelectronic properties of the structure can be tuned and modulated via varying sample parameters such as the diameter of the sphere and the thickness of the Au shell. For the presentation of the experimental results from this study, the transmission spectra of the Au/PS sphere arrays on Si substrate are normalized with the transmittance with respect to the Si substrate. In Figure 3, we show THz transmission spectra for different-sized Au/PS sphere arrays at a fixed Au shell thickness of 10 nm. The transmission spectrum for a 10-nm-thick Au film on Si substrate is shown as a reference. The significant THz absorption occurs for the flat Au/Si film. This implies that a relatively high optical conductance is achieved in the flat film sample. For a normal conducting thin film on an insulating substrate, the electromagnetic energy extinction in the THz region is mainly dominated by free-carrier motion driven by the electric field component of the THz wave when neglecting the magnetic interaction in the film. Therefore, the THz transmission is directly associated to the complex conductivity of the film. It is well known that the Au film on a dielectric substrate experiences a metal-insulator transition in the thickness range of 6 to 7 nm, which results in the abrupt increase in THz absorption (or decrease in THz transmission) with increasing Au film thickness [1]. The THz transmittance of the thin Au/Si film is attributed to the reduced electronic conduction, which arises from the backscattering of the carriers localized in disconnected gold islands [1]. Our experimental results for flat Au/Si films are in line with these experimental findings. However, it should be noted that in [1], the light transmittance for 10-nm-thick Au/Si films was observed at about 0.1 which is smaller than our results. The samples used in the measurements in [1] were produced by thermal evaporation. Such a technique differs significantly from the ion-beam sputtering employed in the sample preparation in the present study. It is known that different techniques to grow Au films on Si wafer can result in different crystallizations of the Au nanoclusters. The better crystallization of the Au nanofilms prepared by thermal evaporation is the main reason why the light transmittance observed in [1] is lower than that measured in this study.

The transmission spectra normalized with respect to the Si substrate for different diameters of Au/PS spheres in the array structures are shown in Figure 3, where the Au shell thickness is fixed at 10 nm. The result for the 10-nm-thick Au film on Si substrate is shown as reference. It can be seen that the transmission spectra of the Au/PS sphere arrays with 10-nm Au shell thickness do not indicate effects similar to the metallic light response. Instead, the intensity of the transmittance for the Au/PS sphere arrays shows a strong dependence on the size of the Au/PS sphere. The THz transmission in a sphere array with 1,000-nm sphere diameter and 10-nm Au shell thickness is pronouncedly larger than that for a flat Au film with the same thickness of the Au layer. In particular, the 200- and 500-nm-diameter sphere arrays are almost transparent within investigated THz bandwidth. This implies that the array samples are optically insulating in the THz regime. Similar results can be observed for array samples with 15-nm Au shell thickness. It is known that in a metal sphere array structure, the electron–electron interaction at the interface between adjacent spheres plays a crucial role in affecting optical conductance and transmittance. The arrangement of the Au/PS spheres in the manner of packing closely to each other can introduce relatively high interface carrier density. Normally, such a density is larger in arrays with smaller Au/PS spheres than in those with larger spheres. A larger interface carrier density corresponds to a stronger interface electronic scattering, which can reduce macroscopic conductivity of the sample. Thus, the optical conductance is smaller in arrays with smaller Au/PS spheres. Hence, the THz transmission increases with decreasing size of the Au/PS spheres in the array structures, as shown in Figure 3.

**Figure 4:**
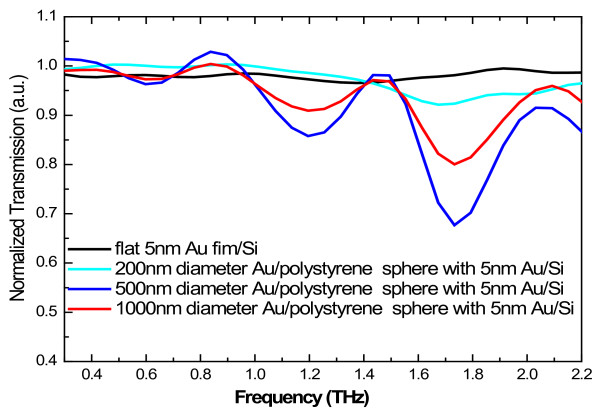
**Normalized transmission spectra for Au/PS sphere arrays with different diameters and fixed 5-nm-thick Au shell.** The result for the 5-nm-thick Au film on Si substrate is shown as reference.

**Figure 5:**
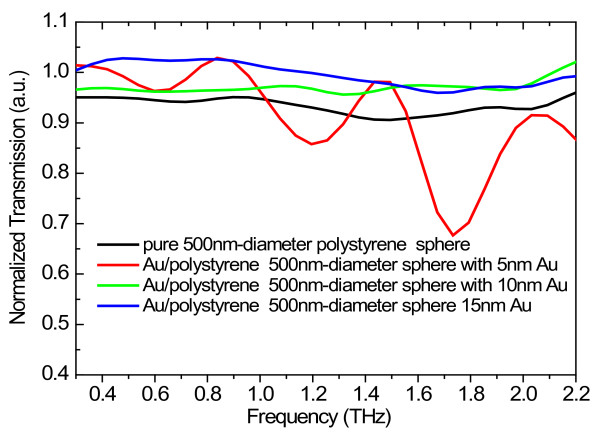
**Normalized transmission spectra for Au/PS sphere arrays with 500-nm diameter and different Au thicknesses.** The result for the pure PS sphere array with a 500-nm sphere diameter on Si substrate is shown as reference.

In Figure 4, the normalized THz transmission spectra for Au/PS sphere arrays with 5-nm-thick gold shell are shown for different diameters of the Au/PS spheres. It is interesting to notice that two absorption peaks appeared at about 1.2 and 1.7 THz which can be clearly observed from the transmission spectra in the samples with 1,000- and 500-nm-diameter spheres. The transmission spectra with a 500-nm-diameter Au/PS sphere in the arrays are shown in Figure 5 for different thicknesses of gold shells. As can be seen from Figure 5, the THz absorption peaks can only be observed clearly for the sample with 5-nm Au shell thickness. The results shown in Figures 4 and 5 indicate that in Au/PS sphere arrays, the critical thickness of Au shell to cause the metal-insulator transition is about 5 nm. The absorption peaks show up in neither pure PS spheres without depositing gold nor flat gold film on Si substrate. This effect suggests that the gold shell on the PS balls is essential in affecting THz response in the array structure. The SPP is known to give rise to the extraordinary high transmission of light radiation through arrays of two-dimensional (2D) subwavelength holes [5,6]. Our observation on THz absorption bands in Au/PS sphere arrays differs from the reported extraordinary THz transmission enhanced in 2D periodical metallic-dielectric structures [7,8]. The lattice constant (about 500 nm) of the Au/PS sphere array samples is far smaller than that in common SPP THz devices (a few tens of microns). The first-order SPP resonant frequency is estimated to be around 170 THz, which is far beyond the spectroscopy range in our measurement.

Different from traditional planar metallic structural arrays on a dielectric substrate, the Au/PS sphere arrays introduce the quantum confinement of electron motion in a radial direction between the barriers from dielectric sphere and air, when the thickness of the gold shell is thin enough. The highly confined electrons in the shell structure's potential well can form quantized electronic states with energy spacing to be in the THz range. This can result in the resonant absorption in THz bandwidth due to inter-subband electronic transition accompanied by the absorption of THz photons. In addition, the periodical structure of the Au shell arrays can also modulate the electronic states and corresponding electron wave functions. For sphere arrays with thin Au shell spheres packed closely to each other, the electron wave function in one sphere can penetrate to other neighboring spheres and form mini-band structures. This can lead to achieve a broadened THz absorption spectrum in the samples. Furthermore, the SPP modes in Au/PS sphere arrays differ significantly from those in Au/substrate film structures. Thus, the coupling between THz electromagnetic field and electrons trapped in the Au shell arrays has some unique features. The results obtained from this study indicate that the strength of coupling between THz light radiation and electrons in the Au/PS sphere array can be efficiently tuned and modulated via varying sample parameters such as the diameter of the Au/PS sphere and the thickness of the Au shell. Consequently, the Au/PS sphere array is a good electronic device in examining photon-induced metal-insulator transition in THz bandwidth.

It should be noted that normally the different curvatures of the nanospheres can lead to different nanocluster distributions on the spheres. This can also affect the THz transmission in the sample structure. The investigation into different cluster modes in different samples with different diameters needs considerable SEM study, and we do not attempt it in the present study. As shown in Figure 4, the THz absorption bands occur at about 1.2 and 1.7 THz at a fixed shell thickness of about 5 nm for sphere diameters from 200 to 1,000 nm. Our very recent theoretical calculations [12] indicate that for hollow nanosphere structures and when the diameter of the sphere is larger than 100 nm, the electronic subband energies degenerate and depend only on the shell thickness *d* via *E*_n_ = *ħ*^2^*π*^2^*n*^2^/2m*d^2^, where *n* is the quantum number and m* is the effective mass for an electron in the gold shell. We know that the THz absorption in the Au/PS nanosphere array is mainly induced by the surface plasmon resonance and the absorption frequency is determined mainly by the surface plasmon frequency. In a quantum structure such as Au nanosphere array, the surface plasmon frequency depends mainly on the energy difference between different electronic subbands. Thus, when the diameter of the Au nanosphere array is larger than 100 nm, the THz absorption frequency depends mainly on the Au shell thickness and depends very little on the diameter of the sample.

## Conclusions

In this study, we have demonstrated clearly that micro/nanostructured periodic Au/PS sphere arrays can tune and modulate strongly the THz light response by varying sample parameters such as the thickness of the gold shell and the diameter of the Au/PS sphere. We have found that the decrease in the sphere size can induce the metal-insulator transition. This is due to the fact that for smaller sized sphere structures, the electron–electron interaction can be enhanced between the adjacent spheres. It is interesting to point out that the THz absorption bands located at about 0.6, 1.2, and 1.7 THz can be observed when the Au shell thickness is about 5 nm. We have tentatively attributed such an extraordinary THz absorption effect to the quantum confinement of the electrons trapped in the gold shell as a potential well between barriers of the dielectric sphere and air. The results obtained from this study suggest that the Au/PS sphere array is a good electronic device in examining photon-induced metal-insulator transition in THz bandwidth. We hope that the experimental findings from this study can shed some light in examining basic physics effects and in applying metal nanosphere array structures as THz devices for various applications.

## Competing interests

The authors declare that they have no competing interests.

## Authors’ contributions

GTD carried out the sample fabrication and SEM study and participated in the THz measurement and manuscript preparation. FHS participated in the THz experiment and drafted the manuscript. WX coordinated the collaborations and carried out the result analysis and the manuscript preparation and revision. CLZ provided the THz measurement facilities and took part in the measurement. WPC initialized and supervised this research work. All authors read and approved the final manuscript.
